# ENPP1 ameliorates vascular calcification via inhibiting the osteogenic transformation of VSMCs and generating PPi

**DOI:** 10.1515/med-2023-0861

**Published:** 2023-12-15

**Authors:** Xiujuan Wu, Shuijuan Shen, Jiaying Wu, Shaorui Wu, Shimi Wang, Feng Di

**Affiliations:** Department of Nephrology, Shaoxing People’s Hospital, Shaoxing 312000, Zhejiang Province, China; Department of Respiratory, Shaoxing People’s Hospital, Yuecheng District, No. 568 Zhongxing North Road, Shaoxing 312000, Zhejiang Province, China

**Keywords:** VSMC, ENPP1, calcification, AAV9, vascular, transformation

## Abstract

This study aims to investigate the impact of ectonucleotide pyrophosphatase/phosphodiesterase 1 (ENPP1) on vascular calcification in rats. The rationale behind studying ENPP1’s role in vascular calcification lies in its potential to modulate calcification processes. Understanding this relationship can offer insights into novel therapeutic avenues for addressing vascular calcification-related disorders. In this experiment, vascular smooth muscle cell (VSMC) calcification was induced using β-glycerophosphoric acid. Subsequently, recombinant AAV9-carrying ENPP1 was introduced into VSMCs to achieve both *in vitro* and *in vivo* overexpression of ENPP1. The findings indicate that ENPP1 overexpression significantly reduces calcium and phosphorus content in the aorta (*P* < 0.05). Alizarin red and von Kossa staining reveal notable reductions in calcium salt deposits in VSMCs and aorta, respectively. Notably, the expression levels of BMP-2, PINP, OC, and BALP were substantially decreased in VSMCs (*P* < 0.05), underscoring ENPP1’s role in impeding osteoblast-like transdifferentiation of VSMCs. Additionally, ENPP1 overexpression led to a significant increase in pyrophosphate (PPi) levels compared to control rats (*P* < 0.05). In conclusion, this study suggests that ENPP1 contributes to alleviating vascular calcification by elevating PPi levels and inhibiting the phenotypic transformation of VSMCs. These findings shed light on the potential therapeutic role of ENPP1 in mitigating vascular calcification-related complications.

## 1 Introduction

Cardiovascular disease stands as a prominent contributor to global mortality, claiming approximately 17.9 million lives in 2016. The majority of these casualties comprise the elderly, a segment particularly susceptible to heart attacks and strokes [1,2]. It has been well established that vascular calcification is closely related to cardiovascular disease and is a critical risk factor and a predictor of long-term prognosis [3]. Hence, the prevention and treatment of vascular calcification have become a focus of clinical cardiovascular disease management. Vascular calcification is not a simple passive process of calcium-phosphate deposition but also occurs through a delicate and organized biological process, including an imbalance between osteochondrogenic signals and anti-calcification factors [4]. Indeed, the imbalance between factors that inhibit and promote calcification is the root cause of vascular calcification [5].

Many factors are involved in vascular and pathological calcifications of cardiovascular structures [6]. Studies show that calcification inhibitors play a major role in initiating the primary mechanism of calcification in the body of patients suffering from vascular calcification [4,7]. Ectonucleotide pyrophosphatase/phosphodiesterase 1 (ENPP1) emerges as a type II transmembrane glycoprotein, displaying dual functionality encompassing nucleotide pyrophosphatase and phosphodiesterase activities. The scrutiny of this gene’s expression primarily delves into its intricate association with the manifestations of mutations in the context of rare diseases [8]. Mutations in the ENPP1 gene can lead to idiopathic infantile arterial calcification, an uncommon autosomal-recessive hereditary disorder in humans, which may have disastrous consequences [9]. Idiopathic infantile arterial calcification is characterized by extensive calcification of medium-sized muscular arteries, which progresses rapidly leading to high mortality from heart failure during infancy [10]. Mutations in the ENPP1 gene also play a role in pseudoxanthoma elasticum, which mainly presents as fragmentation and progressive calcification of elastic fibers in connective tissues [11]. Additionally, ENPP1 plays an important role in the hydrolysis of ATP to produce adenosine monophosphate and pyrophosphate (PPi) [12]. As a synthetase of PPi, ENPP1 directly regulates the levels of PPi *in vivo*.

BALP is considered an important marker of bone formation [13], and its detection is diagnostically important as it is usually upregulated in the calcified artery. PINP is mainly produced during the bone formation process. As the most abundant specific non-collagenous protein secreted by osteoblasts, PINP serves as a sensitive marker of bone formation and bone resorption [14]. PINP can accelerate the formation of calcified nodules, thereby promoting calcium invasion. BMP-2, a member of the TGF-beta superfamily, is a potent bone formation-inducing protein and promotes calcification of adjacent cells primarily through the paracrine pathway and participates in the induction of vascular smooth muscle cell (VSMC) calcification and osteogenic differentiation [15]. OC is a constituent protein of the bone matrix, which regulates bone remodeling and mineralization by altering the activity of osteoblasts and osteoclasts. OC is not only secreted by bones but also upregulated in the calcified VSMCs [16]. It is considered a marker of the transformation of VSMCs to osteoblasts.

In this study, we focused on the role of ENPP1 in inhibiting calcification in both *in vitro* and *in vivo* models and discovered that the overexpression of ENPP1 in either VSMCs or rats significantly inhibited the calcification process. Our findings potentially provide novel ideas for the prevention and treatment of vascular calcification by targeting ENPP1.

## 2 Materials and methods

### 2.1 Cells and molecular biological intervention

Rats were anesthetized with 1% pentobarbital sodium, and the thoracic aorta was quickly cut and repeatedly washed with PBS taken in a 2 mL syringe to remove the coagulation and other tissue blocks adhering to the surface and to clean the surrounding tissue of the blood vessel. Then, it was placed in the enzyme digestion solution, digested in an air bath constant temperature oscillator at 37°C for 45 min, placed in a clean Petri dish for quick shredding, and then transferred to a six-well plate with a compound enzyme solution for digestion for 1 h. Using a 1 mL syringe, the tissue digestion solution was pumped into cells until the cells were basically separated from the tissue fragments. Then, the suspension was filtered with a cell sieve, transferred the suspension together to a 15 mL centrifuge tube, and centrifuged at 1,000 rpm for 5 min to collect the cells. The cells were then suspended in a smooth muscle cell culture medium containing 10% FBS, and seeded into six-well plate for culture. Freshly isolated P0 VSMCs were suspended in a smooth muscle cell culture medium and cultured statically in an incubator for 2 days at 37°C and 5% CO_2_ [11]. The purity of VSMCs was determined based on the positive rate of α-SMA. Smooth muscle cells were divided into three groups, namely, the experimental group, the model group, and the normal group. β-Glycerophosphoric acid (G9422, Sigma, USA) was added to the experimental group and model group at a final concentration of 10 mmol/L. After treatment for 10 days, β-glycerophosphoric acid was replaced with the ordinary culture medium. The target gene ENPP1 virus vector was introduced into the experimental group, an empty virus into the model group, and no intervention was made to the normal group.

### 2.2 Acquisition and amplification of the target gene

PCR amplification is performed using the primers as follows: forward primer: GCGTGAATTCGCCACCATGGAGCGCGACG and reverse primer: GCGTACGCGTGTCTTCTTGGCTGAAGATTGGT. The rat smooth muscle primary cell cDNA is used as the template. Following PCR, 1% agarose gel electrophoresis is used to separate and detect the PCR products, and a DNA purification kit is used to purify the PCR products. The obtained PCR purified products are subjected to *Bam*HI and *Eco*RI digestion and ligated to the pCDH vector to construct the pCDH-ENPP1 recombinant plasmid. The ligation product is transformed into *E. coli* DH-5α competent cells to construct a cloning plasmid. Successfully cloned colonies are selected for restriction endonuclease digestion and DNA sequencing, and the correctly sequenced monoclonal colonies are set up in a centrifuge tube containing ampicillin LB medium and placed in a shaker for 20 h. The bacterial culture suspension is extracted with a High-Purity Plasmid Midiprep Extraction Kit (ZP104-1, Zoman, China), and the obtained plasmid is purified and diluted. At the same time, the vector Pav-fh AAV9 is subjected to the same restriction endonuclease digestion, and the vector is harvested. The constructed virus vector (Pav-fh AAV9-ENPP1) and helper plasmid are extracted in large quantities.

### 2.3 Transfection

The transfection reagent, packaging plasmid, viral vector plasmid, and helper plasmid are mixed in a ratio of 15:2:2:1. After standing at room temperature for 30 min, they are added to HEK293T cells and mixed by shaking. The cells are placed in a 37°C, 5% CO_2_ incubator for 72 h, and then, the virus is collected. Different concentrations of iodixanol are configured, and the virus is purified by density gradient ultracentrifugation. The collected liquid is placed in an ultrafiltration tube to concentrate the virus. About 10 μL of virus solution is aspirated for standardizing the titer and specificity detection. The 96-well plate is infected with HEK293 cells for 24 h to amplify the virus. Adeno-associated virus is treated with proteinase K (5 μg/μL) (P6656; Sigma, USA) to digest the protein coat of the virus, and the virus titer is detected by q-PCR. The normal VSMC of P4 generation is taken, digested, and inoculated in a 6-well plate with 2 × 10^5^ cells per well. When the cell density reaches 70–80%, the cells are infected by the virus. The multiplicity of infection is determined to be 100, and the virus solution is added to VSMC and mixed well for infection at 37°C for 72 h. Cells in the model group are transfected with the empty virus, while no intervention is made to the normal group.

### 2.4 Western blot

Western blot is performed to detect the ENPP1 expression in VSMCs in each group. The cells are rinsed with PBS and digested with trypsin. The cell precipitates are collected, and the lysis buffer is added for protein extraction. Then, the cells are centrifuged, and the supernatant is collected. SDS-PAGE is conducted for 30 min followed by membrane transfer under a steady current of 200 mA. The PVDF membrane is rinsed with deionized water and soaked in the sealing buffer containing 5% skimmed milk powder for 1 h. The sealed PVDF membrane is immersed in the 1× TBST buffer and slowly rinsed on a shaker for 5 min. Then, the membrane is transferred to an incubation box containing the primary antibody and incubated at 4 overnight. The membrane is rinsed with TBST buffer and is transferred to an incubation box containing the secondary antibody. After incubation at room temperature on a shaker for 1 h, the membrane is rinsed with TBST buffer. The PVDF membrane is placed on top of the preservative film and equal volumes of ECL solutions A and B are mixed. The mixture is applied to the surface of the PVDF membrane for chemiluminescence. Antibodies used were as follows: ENPP1 (1:500, ab217368, Abcam, USA), Osterix (1:1,000, A18699, Abclonal, China), Runx2 (1:1,000, A2851, Abclonal, China), α-SMA (1:1,000, A17910, Abclonal, China), GAPDH (1:2,000, AC001, Abclonal, China), and goat anti-rabbit IgG (1:5,000, AS029, Abclonal, China).

### 2.5 PPi detection

The PPi sensor (S6422, Sigma, USA) is redissolved in 50 μL of DMSO to prepare the 200×stock solution. Then, 1 mM standard substance is taken for 1:3 serial dilution. To each well, 50 μL of the standard substance solution is added alongside 50 μL of the sample to be tested. The working solution is prepared, and the PPi sensor is diluted to 1× by using the buffer. To each well, 50 μL of the working solution is added and the cells are incubated at room temperature for 10–30 min. The fluorescence intensity is detected at an excitation wavelength of 316 nm and an emission wavelength of 456 nm.

### 2.6 Alizarin Red S staining

The culture medium is removed and the cells are rinsed twice with PBS. To each well, 500 μL of 4% paraformaldehyde is added to fix the cells for 20 min, which is followed by further rinsing with PBS twice. Then, the cells are incubated with 1% Alizarin Red S (A5533; Sigma, USA) at 37 for 30 min. After rinsing again with PBS three times, the cells are photographed under the microscope.

### 2.7 Phenotypic detection of osteoblast-like cells

The expressions of BMP-2, PINP, OC, and BALP are detected in each group by ELISA (according to the instruction manual). The α-SMA expression in each group is detected by quantitative fluorescence PCR. The primer for α-SMA is F 5′-TCC GGA GCG CAA ATA CTC TGT-3′ and R-5′-CCG GCT TCA TCG TAT TCC TGT-3′. Total RNA extraction from VSMCs is performed using the Trizol reagent (15596026; Invitrogen, USA), followed by reverse transcription into cDNA. The cDNA obtained is subjected to quantitative PCR. The α-SMA expression at the transcriptional level is calculated using the 2^−ΔΔ*C*T^ method.

### 2.8 Animal treatment

Forty-eight male SD rats at 12 weeks of age are purchased from Slacom Experimental Animal Company (Shanghai, China), and 36 rats are randomly chosen and given an intraperitoneal injection of 0.1 μg/mL calcitriol at a dose of 1 μg/kg. The injection volume for each rat is 10 mL/kg. Twelve rats are randomly sacrificed after such injection for 8 consecutive days. The thoracic aorta is harvested for von Kossa staining to determine whether the modeling is successful. After confirming that the model is successfully built, the remaining rats are randomly divided into an experimental group (*n* = 12) and a model group (*n* = 12). Twelve normal rats are selected and given an intraperitoneal injection of normal saline as the normal group. The ENPP1-AAV9 viral fluid is diluted to a titer of 5 × 10^12^ vg/mL with sterile normal saline and quickly injected into the rats of the experimental group via the tail vein. The total quantity of virus injected into each rat is 1 × 10^12^ vg. The viruses (100 μL) are injected in two doses, on days 9 and 16, respectively. In the model group, rats are administered empty AAV9 vectors lacking the target gene, while the normal group is given sterile normal saline. Rats are reared using standard protocols. At weeks 2 and 4 post-injection, six rats from both the experimental and model groups are selected for sacrifice. In parallel, rats from the normal group are sacrificed at week 4. This process yields plasma and thoracic aorta specimens from each respective group.

### 2.9 Determination of indicators and von Kossa staining

The expression of the ENPP1 protein in the thoracic aorta of rats from each group is determined by western blot. The intracellular levels of calcium and phosphorus are determined utilizing colorimetric methods. Vascular calcification is assessed through von Kossa staining. The expression of TNAP mRNA is quantified using real-time quantitative PCR. For the preparation of vascular tissues, a sequential process is undertaken involving fixation, dehydration, clearing, waxing, and embedding into paraffin blocks. Subsequently, the paraffin blocks undergo dewaxing and rehydration through a series of steps: they are successively treated with xylene I for 15 min, followed by xylene II for 15 min, absolute ethyl alcohol I for 5 min, absolute ethyl alcohol II for 5 min, 95% ethanol for 5 min, and 80% ethanol for 5 min. They are then rinsed under running water for 1 min. Von Kossa staining is executed with UV irradiation for 8 min, after which the specimens are rinsed with distilled water for 1 min. A hypo solution is applied for approximately 2 min, followed by counterstaining with HE staining to visualize the nuclei. The process continues with dehydration, clearing, and sealing steps: specimens are sequentially treated with 80% ethanol for 0.5 min, 95% ethanol I for 0.5 min, 0.95% ethanol II for 0.5 min, absolute ethyl alcohol I for 0.5 min, absolute ethyl alcohol II for 0.5 min, and cleared with xylene I for 3 min, and then with xylene II for 3 min. After the previously mentioned treatments, the paraffin blocks are taken out and sealed with neutral balsam. Approximately, to 0.1 g of the vascular tissues 500 μL of the lysis buffer is added. The tissues are homogenized for 15 s. The subsequent steps are the same as the previously mentioned western blot procedure.

### 2.10 Determination of intracellular calcium and phosphorus contents

The working solution is prepared according to the instruction manual and consists of the deionized water, 1 mmol/L calcium standard solution, tissue homogenate supernatant, and working solution II. The optical density (OD) value of each well is detected at a wavelength of 610 nm using a microplate reader, and the intracellular calcium content is calculated based on these values. The intracellular phosphorus content is determined according to the instruction manual (similar to the determination of the intracellular calcium content). In the designated blank, standard, and test wells, 10 μL of distilled water, 1 mmol/L calcium standard solution, and tissue homogenate supernatant are individually introduced. Subsequently, 250 μL of working solution II is added to each well. After thorough mixing and allowing the mixture to stand for 5 min, enzyme-labeled colorimetric analysis is carried out, and the OD value of each well is measured at a wavelength of 610 nm.

### 2.11 Statistical analysis

Statistical analyses are performed using SPSS software. Measurement data obeying a normal distribution are expressed as mean ± SD. Intergroup comparison is conducted using one-way ANOVA. A pairwise comparison of the mean across multiple groups is conducted using the LSD *t*-test. The grayscale value of the target band is analyzed using Image J software. Each experiment is repeated three times, and *P* < 0.05 indicates a significant difference.


**Ethical approval:** This study is approved by the Ethics Committee of Shaoxing People’s Hospital (2020-83).

## 3 Results

### 3.1 ENPP1 overexpression mitigates intracellular calcium contents

**Figure 1:**
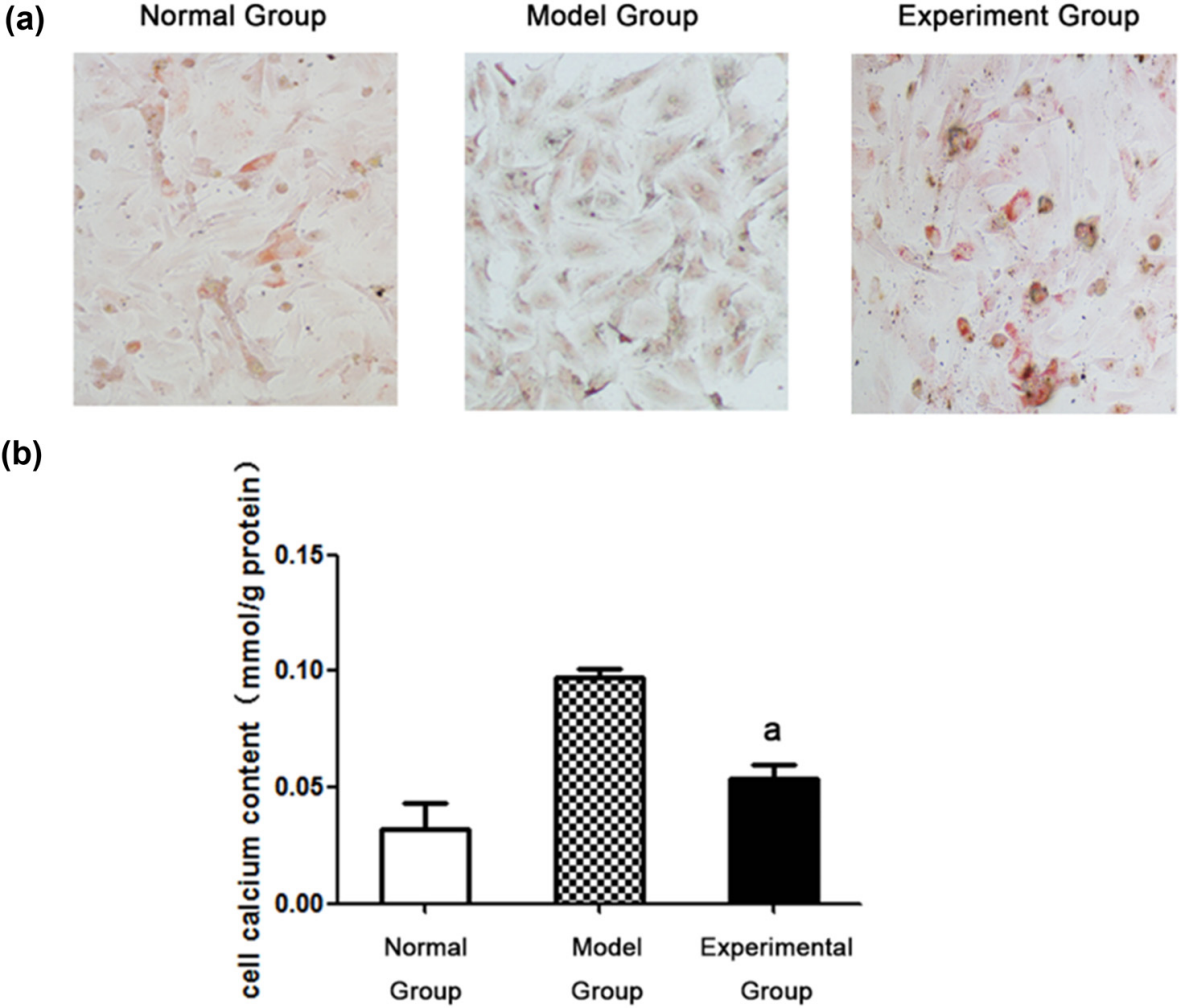
ENPP1 overexpression mitigates intracellular calcium contents: (a) results of the Alizarin red S staining and (b) determination of intracellular calcium contents. A *P* < 0.01 vs the model group.

To characterize the function of the ENPP1 gene, we first transfected glycerophosphate-induced VSMCs with the recombinant ENPP1-containing adenovirus for protein overexpression in VSMCs. Following the successful overexpression of ENPP1, we conducted Alizarin Red S staining to elucidate alterations in VSMC calcification, as depicted in Figure 1(a). The outcomes notably demonstrated that calcium deposition was most pronounced within the model group. However, this severity was markedly attenuated within the experimental group due to the upregulated ENPP1 expression. Within the model group, numerous calcified nodules were evident, accompanied by a profusion of randomly distributed reddish-brown calcium salt particles deposited within the cytoplasm. By contrast, the experimental group shows significantly mitigated calcified nodules and the cytoplasmic deposition of calcium salt particles compared with the model group. Moreover, using a vascular calcification assay, the intracellular calcium content of the experimental group decreased dramatically with the model group, as shown in Figure 1(b). As a result, ENPP1 overexpression effectively reduced intracellular calcium deposition.

### 3.2 ENPP1 regulates the level of calcification-related proteins

**Figure 2:**
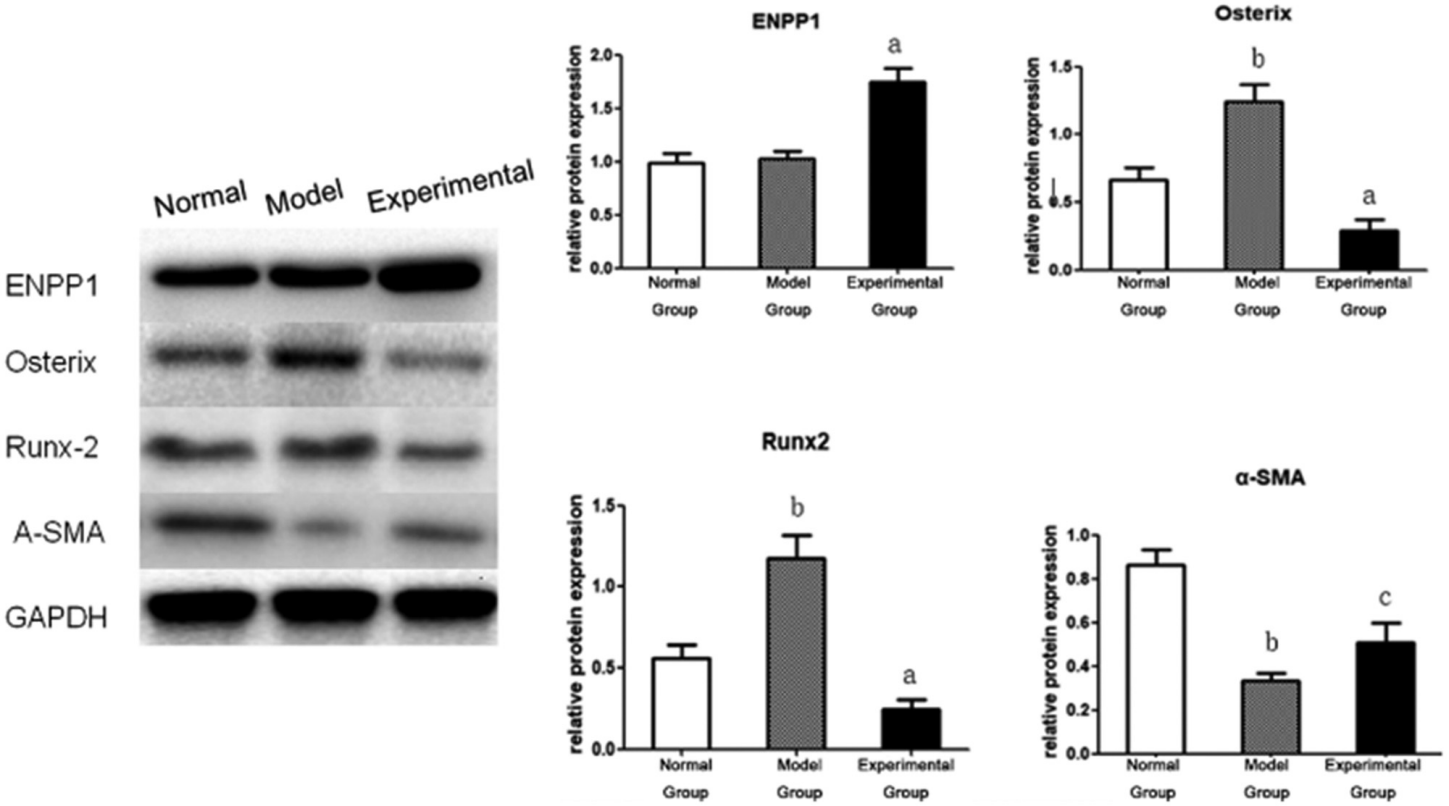
ENPP1 influences the levels of calcification-related proteins. Overexpression of ENPP1 affected the protein levels of Osterix, Runx2, and α-SMA protein in VSMCs. a – *P* < 0.01 vs the model group; c – *P* < 0.05 vs the model group; and b – *P* < 0.01 vs the normal group.

We further explored the influence of ENPP1 on calcification by examining the differential expression of important protein markers, including Osterix, Runx2, and α-SMA, as shown in Figure 2. The western blot shows a substantial decline in the levels in Osterix and Runx2 and an increase in α-SMA compared with the model group. The expression of Osterix and Runx2 proteins in the model group is significantly higher than that in the normal group, while the expression of Osterix and Runx2 proteins in the experimental group is significantly lower than that in the model group. The expression of α-SMA in the model group is lower than that in the normal group, while the expression in the experimental group is higher than that in the model group, with statistical significance (*P* < 0.05). These results suggest that ENPP1 regulated the levels of calcification-related proteins.

### 3.3 ENPP1 overexpression increases the PPi content in VSMC tissues

**Figure 3:**
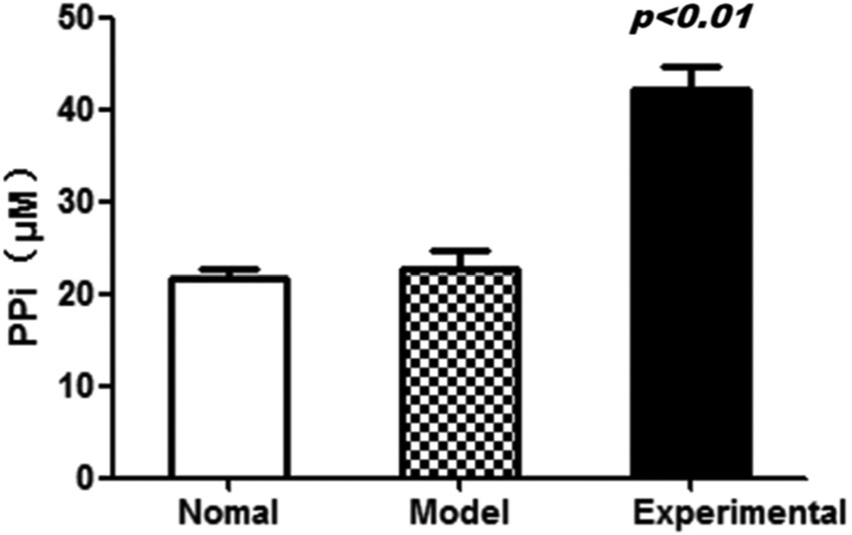
ENPP1 overexpression increases the PPi concentration in VSMC tissues.

To further investigate the influence of ENPP1 overexpression on cell functions, the PPi concentration is quantified in VSMCs using a PPi sensor, as shown in Figure 3. The findings distinctly demonstrate a significant increase in the PPi concentration within the experimental group compared to both the model group and the normal group. This outcome underscores the notable impact of enzyme overexpression on enhancing PPi concentration in VSMCs (*P* < 0.01).

### 3.4 ENPP1 regulates the osteogenic transformation of VSMCs

**Figure 4:**
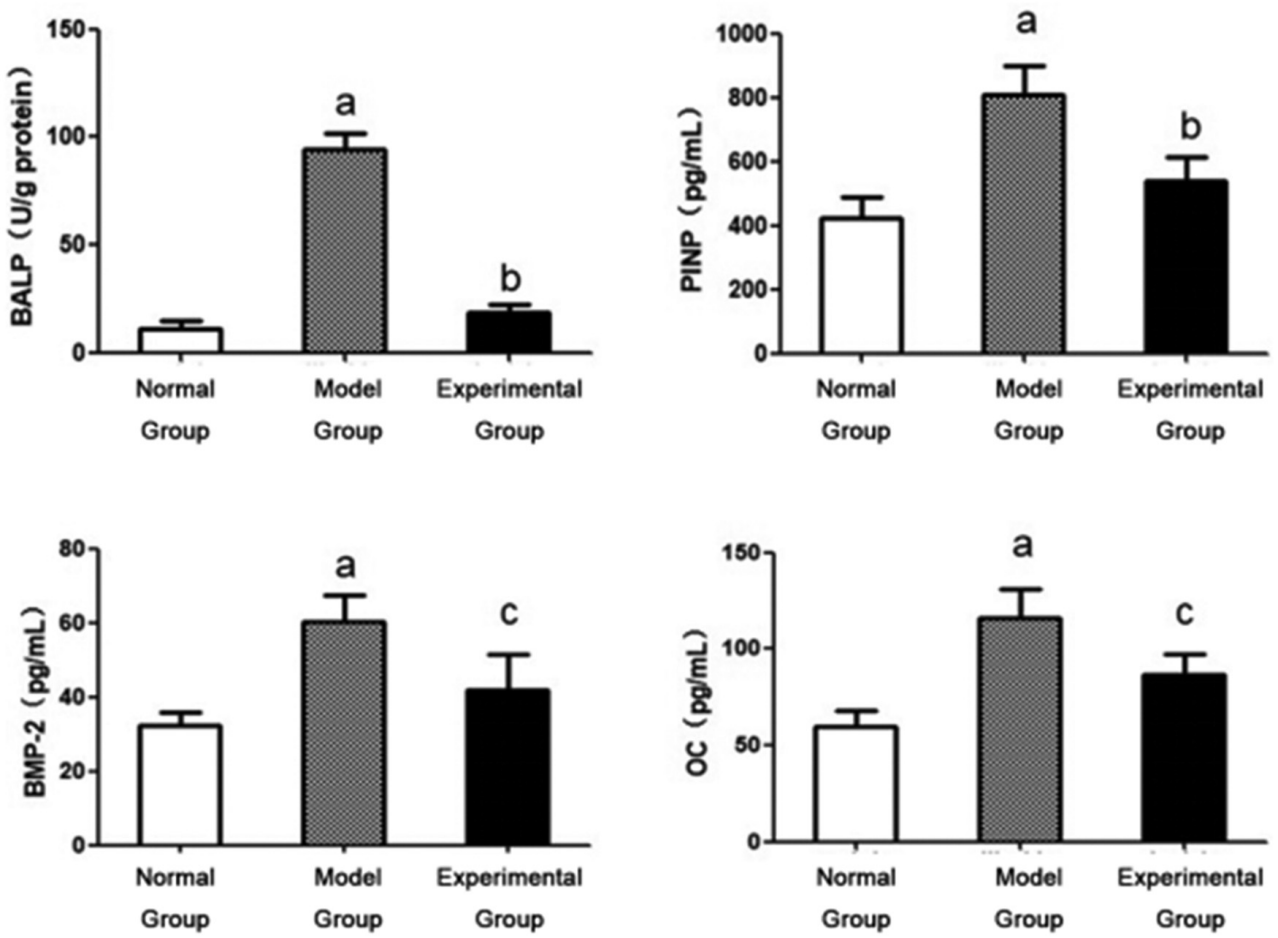
ENPP1 expression regulates osteogenic transformation in VSMCs. Phenotypic markers of the osteogenic transformation of VSMCs in different groups when ENPP1 is overexpressed; a – *P* < 0.01 vs the model group; b – *P* < 0.05 vs the normal group; and c – *P* < 0.01 vs the normal group.

Accumulating evidence from our previous experimental results on the fact that ENPP1 mediated the regulation of calcium and PPi in VSMCs and its effects on the osteogenic transformation of VSMCs, we monitored the changes in the levels of four phenotypic indicators, namely, BMP-2, BALP, PINP, and OC, as shown in Figure 4. The results show that after the induction of calcification by β-glycerophosphate, the levels of these four markers are increased significantly in the model group. However, compared with the model group, the concentrations of these four markers are decreased in the experimental group, respectively, in which the reduction in the BALP concentration is the greatest among the four markers. These results show conclusive evidence to support that ENPP1 is involved in regulating the osteogenic transformation of VSMCs.

### 3.5 ENPP1 inhibits calcification *in vivo*

**Figure 5:**
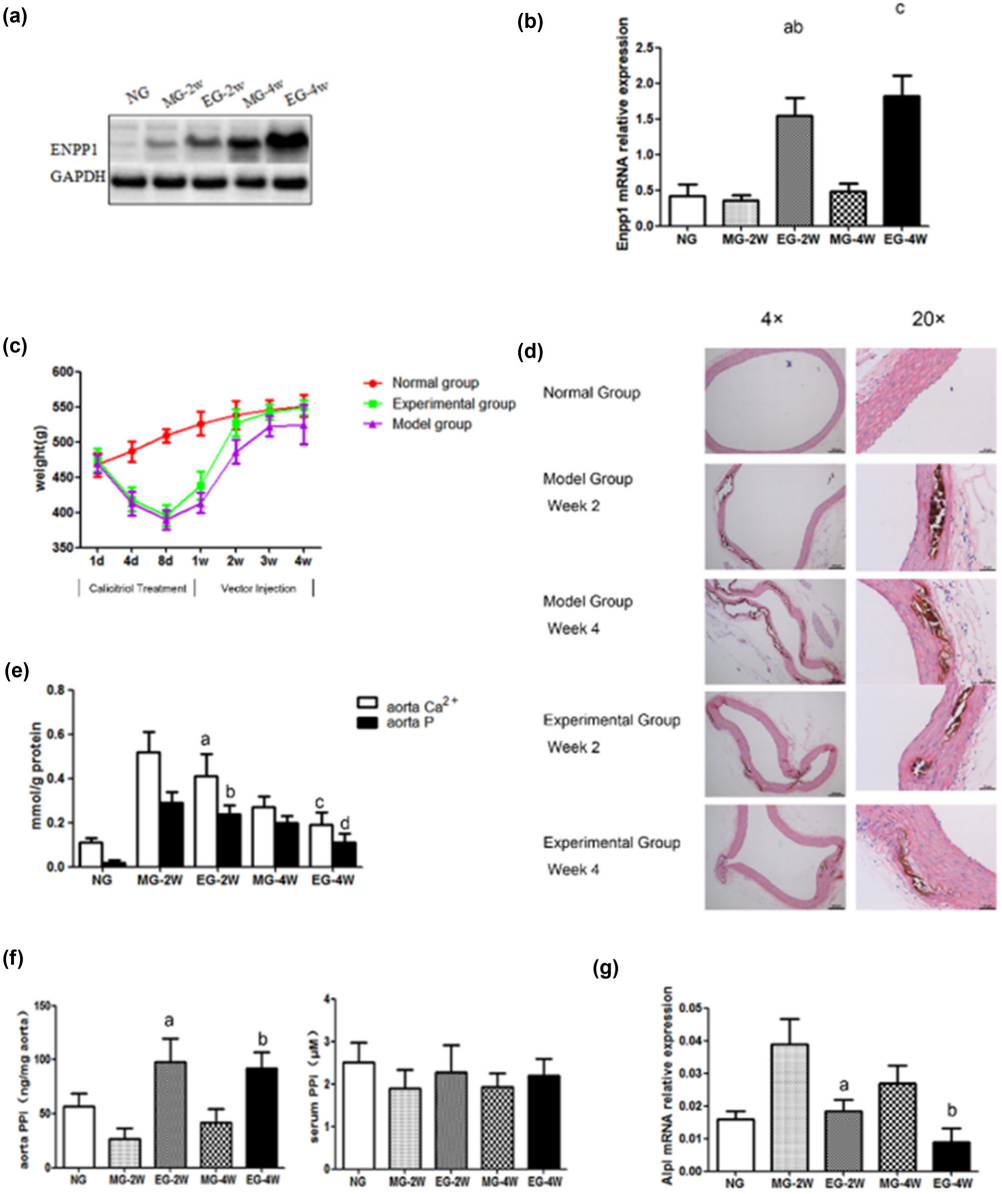
ENPP1 overexpression inhibits calcification *in vivo*: (a) the protein levels increased; (b) mRNA levels increased; a – *P* < 0.01 vs MG-2W; b – *P* < 0.01 vs MG-4W; and c – *P* < 0.05 vs MG-2W; (c) changes in the bodyweights of the rats in different groups when treated with calcitriol; (d) von Kossa staining of the thoracic aorta in rats; (e) determination of intracellular calcium contents; a – *P* < 0.01 vs MG-2W; b – *P* < 0.05 vs EG-4W; c – *P* < 0.05 vs MG-2W; and d – *P* < 0.01 vs EG-4W; (f) intracellular phosphorus content with or without ENPP1 expression; a – *P* < 0.01 vs MG-2W and b – *P* < 0.05 vs MG-4W; and (g) TANP mRNA in the aorta; a – *P* < 0.01 vs MG-2W and b – *P* < 0.05 vs EG-4W.

After verifying the vital role played by ENPP1 in calcium regulation through *in vitro* assays, we further conducted *in vivo* experiments for functional characterization of this gene. The recombinant AAV9-ENPP1 virus vector is injected into the rat via the tail vein. It is shown that at weeks 2 and 4 after injection, the ENPP1 expression in the thoracic aorta of rats in the experimental group is higher than that in the model group. However, after 4 weeks, there is a mild increase in the model group with the normal group, as shown in Figure 5(a). Real-time quantitative PCR indicated a significant increase in the ENPP1 mRNA expression in the thoracic aorta of rats from the experimental group compared with both the model group and the normal group, as shown in Figure 5(b). To assess the influence of ENPP1 overexpression on calcification in rats, we used calcitriol to induce calcification in rats with or without the overexpression of ENPP1. The body weights of rats in different groups are monitored after the administration of calcitriol, as shown in Figure 5(c). It is shown that after discontinuing calcitriol, the body weights of rats in the two groups gradually returned to normal. However, in the experimental group, the body weight bounced back to normal more rapidly than in the model group. Moreover, the body weights of rats in the model group are consistently lower than those of the experimental group after drug administration. At week 2 of drug administration, the difference in the body weight of the two groups was the largest, while the body weight of rats in the normal group steadily increased. Finally, we performed von Kossa staining to elucidate the calcification events in rats, as shown in Figure 5(d). The deposition of brownish calcium salt in the aortic media layer is observed at week 2 in the model group. The calcification is of a higher severity at week 4 than week 2. In the experimental group, the deposition of calcium salt at weeks 2 and 4 is mitigated compared with the model group. In the normal group, there is no calcium salt deposition in the aortic media layer. We also detected the aorta calcium and phosphorus concentrations in the rats, as shown in Figure 5(e). The results are consistent with the *in vitro* data indicating that ENPP1 overexpression decreases the calcium and phosphorus contents. Furthermore, the aorta PPi levels are elevated due to the overexpression of ENPP1, as shown in Figure 5(f). The tissue-nonspecific alkaline phosphatase (TNAP) expression is suppressed as well, as shown in Figure 5(g). These results showed that ENPP1 overexpression inhibited the calcification in rats, which further reinforced the previous *in vitro* data.

## 4 Discussion

In this study, we investigated the relationship between the ENPP1 gene and vascular calcification by transfecting VSMCs both *in vivo* and *in vitro* using an AAV vector. This approach allowed for stable gene expression, and we chose the AAV9 serotype due to its low immunoreactivity and high affinity for VSMCs [17,18]. Our results demonstrated that the overexpression of the ENPP1 gene in VSMCs led to a significant increase in protein expression within these cells. Notably, after inducing VSMC calcification, we observed a substantial reduction in calcium deposition, as evidenced by Alizarin Red S staining. Moreover, intracellular calcium concentrations decreased dramatically, indicating that ENPP1 effectively inhibited *in vitro* calcification of rat VSMCs.

We detected four phenotypic markers of the osteogenic transformation of VSMCs, namely, BMP-2, BALP, PINP, and OC, and the results showed that the levels of all these four markers increased significantly in the model group after the induction of calcification with glycerophosphate as compared with the normal group. However, the levels of these four markers in the experimental group decreased dramatically after the induced overexpression of ENPP1 in VSMCs as compared with the model group. Moreover, we noticed that BALP expression decreased more dramatically compared with the other three phenotypic markers of the osteogenic transformation of VSMCs, indicating that the BALP is a key enzyme regulating bone mineralization at an early stage in osteoblasts, and in turn reflects the differentiation degree and status of osteoblasts.

In the present study, ENPP1 overexpression inhibited four phenotypic markers of the osteogenic transformation of VSMCs, namely, BALP, PINP, BMP-2, and OC, which further inhibited the calcification of VSMCs. The specific mechanism by which ENPP1 inhibits the osteogenic phenotype of VSMCs still remains unclear. It has been shown that aging is related to vascular calcification, and the aging VSMCs usually show an ossification phenotype [19]. ENPP1 may have an anti-aging effect by regulating the expression of the upstream anti-aging gene Klotho [20]. Inhibiting the transformation of VSMCs to the ossification phenotype can lower vascular calcification. The level of α-SMA, as a marker of the contractile phenotype of VSMCs, was determined in the present study and showed that the level of α-SMA in the VSMCs in the experimental group was higher than that of the model group. These findings suggested that ENPP1 could maintain the contractile phenotype of VSMCs while inhibiting its transformation to the ossification phenotype, indicating an important effect of ENPP1 inhibiting vascular calcification.

In our *in vivo* experiments, rats with vascular calcification induced by calcitriol received an injection of recombinant AAV9 carrying the ENPP1 gene. Results revealed upregulated ENPP1 in the rat aorta, leading to a significant decrease in arterial calcium and phosphorus levels, as well as reduced calcium deposition, as shown by von Kossa staining. These findings suggest that ENPP1 overexpression promotes the regression of vascular calcification lesions in rats. Although serum calcium and phosphorus levels in the two groups of rats with ENPP1 overexpression changed only slightly, we observed differences in tissue levels and histological indicators. This indicates that changes in serum levels precede corresponding changes in histological indicators.

Our results showed that ENPP1 overexpression caused a dramatic increase in the serum PPi level of rat VSMCs *in vitro*. It has been reported that PPi can prevent the nucleation of amorphous calcium phosphate and also inhibit the growth of crystals to hydroxyapatite. Moreover, PPi, by binding to the surface of the hydroxyapatite, forms irregular crystals, thereby inhibiting crystal growth and preventing pathological calcification [21,22]. It was also elucidated that vascular calcification is closely related to PPi levels [23], which can also induce changes in gene expression and cell differentiation, and further inhibit the expression of osteopontin and chondrogenesis. Intraperitoneal injection of PPi into rats with uremia significantly reduced the incidence and amount of vascular calcification lesions without affecting bone formation and bone mineralization [24]. In PXE-, GACI-, and ABCC6-deficient mice, ectopic calcification could be significantly mitigated by oral PPi [25]. All of the aforementioned studies have demonstrated that PPi can effectively inhibit vascular calcification. Consistently, in the present study, PPi levels in VSMCs or in the aorta tissues of rats increased significantly after ENPP1 overexpression. The reduction in Pi levels in tissues implicated the key role played by ENPP1 in the regulation of PPi/Pi, which in turn controls the deposition of bone minerals. However, the influence of ENPP1 overexpression on the serum PPi was not significantly different, indicating that the local PPi concentration in the tissues played a more critical role in the regulation of vascular calcification than the PPi level in circulation.

ENPP1, along with the TNAP, regulates the Pi/PPi ratio [26,27]. Contrary to the function of ENPP1, TNAP catalyzes the generation of two molecules of inorganic phosphorus from the PPi to promote calcification. To determine the influence of ENPP1 overexpression on TNAP, we also detected the mRNA expression levels of TNAP. The results showed that the ENPP1 overexpression led to an inhibited ALPL mRNA expression, which further influenced the level and activity of TNAP directly or indirectly, thereby further reducing PPi degradation. As a result, the level of PPi for inhibiting calcification was maintained, which was favorable for inhibiting the generation of substrate Pi for the nucleation of hydroxyapatite. As the PPi/Pi ratio increased, vascular calcification was inhibited. Although TNAP plays an important role in the metabolism of PPi, the synthetic rate of PPi is ten times the hydrolysis rate [28]. Therefore, ENPP1 plays a decisive role in controlling the extracellular level of PPi. BALP as a bone formation marker in our cell experiment is one of the three secondary isozymes of TNAP, and like TNAP, BALP also hydrolyzes PPi. However, its function is just the opposite of ENPP1, and BALP can be inhibited by ENPP1.

## 5 Conclusion

Taken together, ENPP1 can inhibit VSMC calcification and promote the regression of vascular calcification in rats. ENPP1 may work by promoting the generation of PPi, regulating the PPi/Pi ratio, and inhibiting the transformation of the contractile phenotype of VSMCs to the ossification phenotype.

## Abbreviations

ENPP1: ectonucleotide pyrophosphatase/phosphodiesterase 1
PPi: pyrophosphate
TNAP: tissue-nonspecific alkaline phosphatase
VSMC: vascular smooth muscle cell
